# Mutual Interactions among Exercise, Sport Supplements and Microbiota

**DOI:** 10.3390/nu12010017

**Published:** 2019-12-20

**Authors:** Sabrina Donati Zeppa, Deborah Agostini, Marco Gervasi, Giosuè Annibalini, Stefano Amatori, Fabio Ferrini, Davide Sisti, Giovanni Piccoli, Elena Barbieri, Piero Sestili, Vilberto Stocchi

**Affiliations:** Department of Biomolecular Sciences, University of Urbino Carlo Bo, 61029 (PU) Urbino, Italy; marco.gervasi@uniurb.it (M.G.); giosue.annibalini@uniurb.it (G.A.); s.amatori1@campus.uniurb.it (S.A.); f.ferrini2@campus.uniurb.it (F.F.); davide.sisti@uniurb.it (D.S.); giovanni.piccoli@uniurb.it (G.P.); elena.barbieri@uniurb.it (E.B.); piero.sestili@uniurb.it (P.S.); vilberto.stocchi@uniurb.it (V.S.)

**Keywords:** gut microbiota, physical exercise, sport supplements, training adaptation, performance, health

## Abstract

The adult gut microbiota contains trillions of microorganisms of thousands of different species. Only one third of gut microbiota are common to most people; the rest are specific and contribute to enhancing genetic variation. Gut microorganisms significantly affect host nutrition, metabolic function, immune system, and redox levels, and may be modulated by several environmental conditions, including physical activity and exercise. Microbiota also act like an endocrine organ and is sensitive to the homeostatic and physiological changes associated with training; in turn, exercise has been demonstrated to increase microbiota diversity, consequently improving the metabolic profile and immunological responses. On the other side, adaptation to exercise might be influenced by the individual gut microbiota that regulates the energetic balance and participates to the control of inflammatory, redox, and hydration status. Intense endurance exercise causes physiological and biochemical demands, and requires adequate measures to counteract oxidative stress, intestinal permeability, electrolyte imbalance, glycogen depletion, frequent upper respiratory tract infections, systemic inflammation and immune responses. Microbiota could be an important tool to improve overall general health, performance, and energy availability while controlling inflammation and redox levels in endurance athletes. The relationship among gut microbiota, general health, training adaptation and performance, along with a focus on sport supplements which are known to exert some influence on the microbiota, will be discussed.

## 1. Introduction

The human adult gut microbiota contains trillions of microorganisms belonging to thousands of different species, and it is recognized as having a pivotal role in overall health and disease of the host [1]. The normal human gut microbiota comprises of two major phyla, namely *Bacteroidetes* and *Firmicutes*, and of less present phyla, such as *Actinobacteria* and *Verrucomicrobia* [2]. These microorganisms inhabit the human gut and form a complex community interacting with each other and with the host. Several environmental factors shape the gut microbiota; even if a dominant role is played by diet, physical exercise is also emerging as an important modulator [3,4]. Dietary composition influences the composition of gut microbiota: Diets with a low amount of fiber and rich in refined carbohydrates and fat cause a reduction in community diversity with alterations in structure and activity of the microorganisms [5,6,7]. Apart from diet, exercise is considered as one of the main environmental factors possibly influencing gut microbiota composition in animals and humans [3,8,9].

Microbiota acts as an endocrine organ, and the changes in its status induced by exercise have been correlated with modifications in host physiology, metabolism, immunity, and behaviour [10,11,12]. The effects of exercise on gut microbial microorganisms depends on its intensity and timing as well as on the anamnestic characteristics of the subject enrolled. The microbiota can also influence muscle mass, as reported by Ticinesi et al. [13]. Sarcopenia rat models exhibit a reduction of several taxa with anti-inflammatory and pro-anabolic effects in gut microbiota [14]. To compensate for their increased energy consumption and maximize their adaptation to physical loads, athletes must adopt adequate dietary habits, which increasingly include a broad range of sports nutritional supplements. These supplements could also exert specific effects on the microbiota composition, metabolism, and functionality, thus representing another, yet underestimated, variable.

In this review, we analyze the effects of mutual interactions between exercise, sport dietary supplements, and microbiota on the overall general health and performance of athletes.

## 2. Functions of Microbiota

Metabolomics, metagenomics, transcriptomics, proteomics, and glycomics have significantly contributed to clarifying the gut microbiome composition—which is now almost completely known—and its association with human health [15]. The MetaHIT and Microbiome Project (HMP) consortia worked hard on determining the baseline structure and function of microbiomes, producing a complete list of species and related genes. However, diversities at sub-species and strain levels are still emerging, and several microbial genes remain functionally uncharacterized [8].

The major phyla included in the human microbiome are *Firmicutes* and *Bacteroidetes*, but also a large number of minor phyla (i.e., *Actinobacteria*, *Verrucomicrobia,* and *Proteobacteria*) showing high metabolic activity are present [16,17]. *Bacteroides*, *Bifidobacterium*, *Streptococcus*, *Enterobacteriaceae*, *Enterococcus*, *Clostridium*, *Lactobacillus*, and *Ruminococcus* are the predominant luminal microorganisms, while *Clostridium*, *Lactobacillus*, *Enterococcus*, and *Akkermansia* are the predominant mucosa and mucus-associated genera.

The gut microbiota reflects the health status of the colonized organisms since it is involved in nutrient and drug metabolism, intestinal barrier function, and protection from colonization of pathogens, working collaboratively with the immune system. It participates in the signaling network and is involved in barrier functions and in the maintenance of its homeostasis [18].

Some features have been associated with healthy gut microbiota: The *Firmicutes:Bacteroidetes* ratio has been widely discussed. The *Firmicutes:Bacteroidetes* ratio undergoes an increase from birth to adulthood and is further altered with advanced age [19]. Ley et al. [20] reported a higher relative proportion of *Bacteroidetes* in lean people compared to obese ones, and found an increase in this phyla to be associated with weight loss, suggesting that obesity has a microbial component. Other authors failed to find weight loss diet-dependent significant changes in the proportion of *Bacteroidetes* in obese subjects, but evidenced a reduction in a group of butyrate-producing *Firmicutes* [21]. No association between BMI and *Firmicutes:Bacteroidetes* ratio or taxonomic composition in adults was found [22], while Sze et al. [23] detected only a weak association between microbial communities and obesity, evidencing that detection is confounded by a large interpersonal variation in microbiota composition. Recently, a positive association between *Bacteroides fragilis* and childhood obesity and a decrease in *Bacteroidetes phylum* related to high BMI were reported. For the *Firmicutes phylum,* a positive correlation with weight gain was found, and some *Firmicutes* species are associated with adipose tissue storage, even if further well-designed studies on this topic are required [24]. It has also been demonstrated that a low amount of *Proteobacteria* together with a high abundance of *Bacteroidetes*, *Prevotella*, and *Ruminococcus* is favourable for health [25].

The most important nutrient source for gut microbiota is dietary indigestible carbohydrates, whose fermentation produces acetate, propionate, and butyrate—named short-chain fatty acids (SCFAs)—the gases H_2_ and CO_2_, ammonia, amines, phenols, and energy, which the bacteria use for growth and the maintenance of cellular function [26].

Bacteria of the genus *Bacteroides* are known to possess very large numbers of genes that encode carbohydrate-active enzymes, such as glycosyl transferases, glycoside hydrolases, and polysaccharide lyases, performing carbohydrate fermentation [27]. The main SCFAs produced are acetic acid (C2), propionic acid (C3), and butyric acid (C4) (ratio 60:20:20), and in lower amounts, lactic acid (mainly L-lactate), 2-hydroxy propanoic acid, valeric, and caproic acids. Acetic and propionic acids are mainly absorbed from the colonic lumen, while butyric acid is the substrate of colonocytes. Inflammatory bowel disease, irritable bowel syndrome, cancer, and other pathologies are strictly related to SCFAs production [28].

Proteolysis is also affected by gut microbiota since several bacteria produce their proteinases and peptidases that work in synergy with human ones; furthermore, histamine is produced from L-histidine and γ-amino butyric acid from glutamate, and important antimicrobial peptides (bacteriocins) are generated [29,30]. Gut microbiota greatly contributes to the synthesis of vitamin K, biotin, folic acid, thiamine, glycans, amino acids, and conjugated linoleic acid (CLA) that possess several health-protective activities [31,32]. The lipid metabolism is influenced by gut microbiota, promoting the lipoprotein lipase activity in adipocytes and alteration of bile acid profile [33]. The up-regulation of the colipase by *Bacteroides thetaiotaomicron* augments the efficiency of lipid digestion [34]. Furthermore, some species, such as *Escherichia coli* and *Bacteroides intestinalis* and *Bacteroides fragilis*, transform the primary bile acids into the secondary bile acids in the human colon [35]. Baars et al. [36], reported that the sex-specific differences in lipid metabolism are modulated via the gut microbiota in mice and bile acids possibly play a role in the crosstalk between the microbiome and sex-specific regulation of lipid metabolism.

The gut microbiota contributes to the metabolization of polyphenol compounds, assumed with a diet rich in plants, fruit, and plant-derived products, such as cocoa, berries, wine and tea. An important role of gut microbiota on xenobiotic and drug metabolism, therefore in the outcome of pharmacological therapies, has also been reported.

Microbiota composition shows a certain degree of inter-individual differences that can be due to microbiome intrinsic factors (i.e., age-dependent state, compositional state) and microbiome extrinsic factors, such as host extrinsic factors (i.e., lifestyle, dietary habits, physical activity), host intrinsic factors (i.e., genetics), and environmental factors (i.e., vertical maternal transmission) [8]. With regard to age differences, the intestinal composition is greatly determined in childhood and reaches adult shape at three-years of age, influencing immune system functionality [37,38,39]. In adulthood, the human gut microorganisms remain relatively stable over time, exhibiting resilience to disruptors such as stress and acute diseases [40,41], even if some evidence shows that distinct compositional types of the gut microbiome, the enterotypes, can be associated with different responses to perturbations [42,43]. For example, *Prevotella copri* and *Bacteroides vulgatus*, that characterize different enterotypes, are involved in insulin resistance [44], and modification on glucose metabolism following a dietary intervention can be influenced by the *Prevotella:Bacteroides* ratio [8,45]. In the older age, species richness and diversity of the microbiome decreases and inter-individual variability increases, while the resilience to perturbations is reduced [46,47].

## 3. The Relationships between Microbiota and Exercise

The gut bacterial population is modulated by gender, genetics, age, and ethnicity, i.e., non-modifiable factors, and by modifiable factors such as host health, physical activity, diet, and eventual antibiotic therapies. Campbell et al. [48] showed that exercise manifested a unique effect on the microbiome independent of diet. Physical exercise is linked with the positive modulation of gut microbiota biodiversity; the potential mechanisms by which exercise might alter the gut microbiome have been investigated in animal and human studies.

The modifications of gut microbiota induced by physical exercise are due to the gut transit time [49], the modification of the bile acids profile [50], the production of SCFAs via AMPK activation [9,51], the modulation of the Toll-Like Receptors (TLRs) signaling pathway [52,53], immunoglobulin A (IgA) [54], the number of B and CD4+ T cells, and finally, to the weight loss [55].

Furthermore, regular exercise reduces the heat shock proteins’ response to heat stress, preventing the breakdown of tight junction proteins between intestine epithelial cells. Thus, the exercise represents a hormetic stressor to the gut that stimulates beneficial adaptations and improves the long-term resilience of the gut barrier [56].

### 3.1. Effect of Exercise on Gut Microbiota Metabolism

Several factors should be considered when analyzing the impact of exercise on microbiota metabolite production, such as age, body fat, and training status of subjects. Exercise effects are amplified and start early in life; alongside an increase in *Bacteroidetes* and a decrease in *Firmicutes*, physical activity promotes, more in the young than in adults, an increase in lean body mass through an adaptation of host metabolism mediated by bacteria composition [57]. Furthermore, microbial species promoted by exercise at this age favour optimal brain function development [58]. In a study analyzing active and sedentary women under 40 years of age after exercise training, the changes in several bacterial taxa were significantly correlated with body mass index (BMI) [59]. Even if the microbiota composition of all the participants was changed for a period following exercise, species with known anti-inflammatory properties and the ability to produce SCFAs were higher in lean subjects [59]. Athlete microbiomes have been found to contain distinct microbial compositions defined by the elevated abundance of *Veillonellaceae*, *Bacteroidetes*, *Prevotella*, *Methanobevibacter,* and *Akkermansia* [60]. The abundance of taxa involved in energy and carbohydrate metabolism, such as *Prevotella* and *Methanobrevibacter smithii*, were found to be significantly higher in professional than amateur cyclists, and were correlated with the frequency of training [61]. Christensen et al. [62] demonstrated in overweight adults, following a fiber and whole-grain-rich diet for six weeks, that *Prevotella* abundance is predictive of weight loss, suggesting that enterotype should be considered in personalized nutritional strategies to counteract obesity. SCFA production, in particular butyrate, an important marker of gut health, increases after exercise in humans [9]. In fact, women performing three hours of exercise or more per week increased the relative abundance of butyrate-producing taxa, and in particular some species such as *Faecalibacterium prausnitzii*, *Roseburia hominis*, and *Akkermansia muciniphila*, as well as the *Coprococcus* genus, compared to sedentary counterparts [59]. These species have been related to health-promoting effects. A high abundance of *A. muciniphila* has been previously described in the microbiota of athletes, while low levels have been linked to metabolic disorders (obesity, metabolic syndrome, and type II diabetes) in patients with inflammatory bowel disease (IBD) [63,64]. Furthermore, the exercise may positively impact the gut mucus layer, an important substrate for mucosa-associated bacteria such as *A. muciniphila*. Butyrate produced by both *R. hominis* and *F. prausnitzii* has a beneficial effect on health with a positive impact on intestinal function and lipid metabolism [65,66]. *F. prausnitzii* also produces metabolites with anti-inflammatory action [67]. The *Coprococcus* genus, a butyrate-producing genus, was more abundant in active women, promoting some exercise-related health effects [59]. In another study comparing lean and obese adults participating in a six-week supervised endurance exercise program under dietary control, an increase in butyrate-producing taxa was found only in lean subjects. Furthermore, lean adults had an increase in *Faecalibacterium* species, while obese adults had a decrease, and *Bacteroides* species had an opposite trend, confirming the influence of BMI [9]. Interestingly, the abundance of butyrate-producing taxa was higher in individuals with greater levels of aerobic fitness after normalisation for BMI, diet, and age [68].

Physical activity based on long-distance running led to significant increase in the *Coriobacteriaceae* family. Notably, this bacterial family—along with others—is involved in the conversion of scarcely absorbed dietary polyphenols to bioavailable and bioactive derivatives [69], as well as in the metabolism of bile salts and steroids, in particular the steroid aldosterone 18-glucuronide. This metabolite of aldosterone exerts many important functions, such as cell signaling, fuel and energy storage, and membrane integrity and stability [70].

During and after exercise, significant amounts of lactate are released into the blood. In particular, lactate has an important role in endurance performance, inasmuch as it is used as fuel from several organs and tissues. The more these organs and tissues “learn” to use lactate as a substrate, the more the performance improves [71].

Recently, Scheiman et al. [60] demonstrated that systemic lactate can cross the gut barrier into the lumen and then can be converted into propionate by the genus *Veillonella*. These authors have recently reported that *Veillonella* abundance increases in the gut microbiota and its Methylmalonyl-CoA pathway is overexpressed post-exercise; furthermore, they demonstrated that in mice, *Veillonella* inoculation improved treadmill performance, which was also improved by propionate administered via intracolonic infusion. Thus, these studies suggest that the modulation of SCFA production by gut microbiota affects energy metabolism during exercise and hence contributes to exercise-induced adaptation. These products of microbiota fermentation can also be used as sources of energy in the liver and muscle cells to improve endurance performance by maintaining glycaemia over time [72,73].

Again, it is important to highlight that human gut microbiota composition remains relatively stable over time, exhibiting resilience to perturbations [16], or returning totally or partially to the previous composition after stimulus cessation [74]. The resilience to perturbations implies that positive changes in exercise and dietary habits aimed at inducing positive effects on the microbiome need to be maintained for a long period to be effective. A summary of studies assessing the impact of physical activity or exercise intervention on the human gut microbiome is detailed in Table 1 [9,11,12,48,57,59,60,61,63,68,70,75,76,77,78,79,80,81,82,83,84,85,86,87,88,89,90,91,92,93].

### 3.2. Microbiota and Exercise-Induced Stress

Alongside the positive effects of physical activity on gut microbiota documented by the studies previously described, unfavorable changes in host physiology have been described. High-intensity exercise not supported by an adequate training level, or overtraining syndrome, may be a stressor for the organism and may also have a negative effect on gut microbiota [13,94]. This is in agreement with the hormetic effects of reactive oxidative species (ROS) generated by exercise and by specific commensal gut microbiota [95,96,97,98,99], that at physiologically normal levels promote positive effects by participating in specific signaling pathways; while at higher concentrations, attained in the presence of toxic or pathological conditions, exert detrimental effects. Indeed, a recent key discovery in this direction was the demonstration that several species of human commensal gut bacteria are capable of generating low levels of ROS, which may contribute to sharpening the beneficial effects promoted by normal microbiota [99,100]. Modulation of ROS-mediated signaling may occur during quantitative or qualitative changes in the composition of gut microbiota following rapid dietary changes, antibiotic therapy, or probiotic intake [99]: These modulations may consequently modify and/or reduce the generally positive influence of microbiota-derived ROS. Changing from a “beneficial” to a “detrimental” response will depend on many variables, which also include the duration and intensity of muscle effort and the overall antioxidant status of the organism. In this scenario, it is important to consider the novel role of microbiota, which is capable of consistently modulating the diverging effects of ROS.

The basic principle in training theory is to push athletes to increase their training volume and intensity to the limits to maximize their performance. High-intensity exercise, especially if not proportional to training level, has a profound impact on oxidative stress, muscle damage, systemic inflammation, and immune responses [101]. Excessive exercise and inadequate recovery cause physical and psychological stress that are interrelated and lead to performance decline, fatigue, insomnia, anxiousness, inflammation, and immunosuppression [102].

From the gut microbiota perspective, prolonged exercise determines an increased intestinal permeability, altering the gut-barrier function and promoting bacterial translocation from the colon [103,104]. High-intensity exercise can cause changes in the immune response by reducing the gastrointestinal (GI) blood flow, thus increasing tissue hyperthermia and permeability of the GI epithelial wall. Research on exercise and barrier function suggests that the more strenuous the exercise, the greater the barrier disruption. GI illness symptoms, including abdominal pain, nausea, and diarrhoea, might affect 70% of athletes after strenuous exercises, and the frequency is higher in elite athletes than in recreational exercisers [105]. Excessive exercise induces a stress that increases intestinal permeability, which might lead bacteria and their toxic products, including microbiota-derived LPS, to enter into the bloodstream and activate systemic inflammation [106]. The translocated LPS activates TLRs, promoting NF-kB-pathway activation and inflammatory cytokine production, which can eventually result in endotoxemia [13]. These pro-inflammatory cytokines also increase the intestinal permeability through tight junction opening [107].

Exhaustive and acute endurance exercise has been demonstrated to induce apoptosis and altered permeability in a murine model [108]. Karl et al. [109] showed that an intense military training resulted in increased intestinal permeability concomitant with changes in intestinal microbiota composition (increased abundance of less dominant taxa and decreased abundance of more dominant taxa such as *Bacteroides*) and markers of inflammation. A half marathon race in amateur athletes changed the fecal microbiome functionality, causing a pro-inflammatory profile [70].

In a study in mice on endurance swimming time, Hsu et al. [81] demonstrated a relationship between performance and antioxidant activity of gut microbiota, suggesting that “gut microbiota status could be crucial for exercise performance and its potential action linked with the antioxidant enzyme system in athletes”.

Alongside single species activity and the effects on exercise response, performance could be affected by the ecological community as a whole. Indeed, the gut microbiota has a key role in controlling oxidative stress, inflammatory responses, metabolism, and energy expenditure during intense exercise.

Exercise alters the turnover of molecules involved in metabolic patterns and stimulates the release of neuroendocrine hormones interacting with the gut directly or indirectly through the immune system [110]. Alongside the adaptation of gut microbiota to exercise training, intestinal microbiota’s influence on exercise performance should be considered.

Upper respiratory tract infections (URTI) represent a common problem in sport [105,111]. URTI frequency is higher during intense training or competition, especially in individual sports such as triathlons and marathons, and lower in team sports [112]. Moreover, travel, stress, and low energy can increase URTI risk in athletes [113]. URTI can greatly affect performance, reducing muscular strength and coordination, aerobic capacity, and concentration. Due to the role of gut microbiota in programming the mucosal immune system, it can affect the respiratory tract susceptibility to infections [111]. Indeed, gut microorganisms are able to regulate mucosal sites distal from the intestine through the common mucosal immune system induction of immunoglobulins [114].

The notion that intestinal microbiota influence respiratory health is also highlighted by recent studies demonstrating that gut health-promoting products, such as probiotics and prebiotics, might reduce susceptibility to URTI [115,116], as detailed in the Section 5.2.

The activation of sympatho-adrenomedullary and the hypothalamus–pituitary–adrenal axis during exercise leads to catecholamine and glucocorticoid release [117]; furthermore, the autonomic nervous system increases neurotransmitters in peripheral tissues, such as the GI tract, and interacting with the enteric nervous system, regulates the passage of material through the GI tract [118]. The gut itself produces hormones such as gamma aminobutyric acid and neuropeptide Y [119], and microbiota are able to secrete molecules such as SCFAs and tryptophan [120,121] which can, in turn, modulate inhibitory and excitatory neurotransmitters and neurotransmitter-like molecules [122]. Dopamine production during exercise-induced stress is released in the GI tract depending on the levels of tyrosine, the type of stress, sex, and the presence of enteric bacteria capable of producing this non-essential amino acid [123]. The release of stress hormones can result in increased intestinal permeability.

Fatigue is an important limiting factor during strenuous exercise; it is a complex phenomenon resulting from muscle exhaustion and modification in the central nervous system linked to increased serotonin levels. The presence of elevated cerebral serotonin levels observed in rats under fatigue [124] is the basis of a well-accepted theory to account for the onset or increase of central fatigue in humans. Indeed, during sustained exercise, increased brain uptake of the serotonin precursor tryptophan has been observed in humans [125,126]. This theory has recently been bolstered by Kavanagh et al. [127], whose study based on paroxetine administration in humans demonstrated the influence of serotonin availability in increasing central fatigue under prolonged maximal contractions. It is also worth noting that dopamine is an important factor involved in the motivation of athletes [128]. The larger amount of secreted serotonin is produced by intestinal enterochromaffin cells, but a significant proportion is produced through gut microbiota synthesis [129]. As such, when dopamine levels decrease together with increased serotonin, it may trigger a state of increasing tiredness during exhaustive exercise, and be involved in fatigue insurgence during exercise impairing performance [130]. The maintenance of a healthy microbiota can also influence an athlete’s mood, motivation, inflammation, and susceptibility to infections: All of these effects improve performance and reduce fatigue onset and perception.

As above-mentioned, diet can greatly affect microbiota composition; the America Dietetic Association suggest a moderate to high intake of animal proteins to restore muscle, a high amount of simple carbohydrates to maintain glucose homeostasis, and a moderate intake of fats and fibers to reduce GI problems in athletes [131]. In addition, animal proteins can negatively affect microbiota through the production of toxic by-products, while a high simple carbohydrate diet leads to a reduction of beneficial SCFAs and immune function [132]. Furthermore, insufficient fibers and resistant starch can cause a reduction in microbiota diversity and functionality [133] (see also 5.3 for the effects of protein intake and supplementation).

## 4. Effects of Dietary Supplementation on Gut Microbiota Profiles: Can They Affect Performance?

The composition of the diet and food-associated microbes in athletes can influence the composition of gut microorganisms able to improve energy metabolism, oxidative stress, and systemic inflammation status, and the microbial species that produce toxic metabolites from protein degradation [134].

With this in mind, we will describe and discuss the supplements that have been reported to have some effects on microbiota composition, and discuss what the implications of such interactions may be for physical exercise and sport practice. To this end, we narrowed our search to the agents and supplements that have been recognized as useful and beneficial to athletes’ health and/or performance by Close et al. [135] (Table 2), and their effects on microbiota are also summarized in Table 3.

## 5. Sports Supplements with Strong Evidence of Interactions with Microbiota

### 5.1. Antioxidants (Polyphenols)

It is known that exhaustive exercise may lead to a strong increase in ROS, which may exceed the endogenous antioxidant capacity and cause severe oxidative damage, muscle weakness, and fatigue. Furthermore, muscle injuries—via cytokine production—recruit and activate neutrophils and macrophages which in turn can produce excessive and/or additional ROS levels [136]. The frequent incidence of the above situations in athletes represents the basis for today’s popularity of antioxidant-containing sport nutrition supplements. A key category among antioxidants are polyphenols, a wide group of natural plant-derived compounds [137], commonly included in supplements intended for sport use (i.e., berry extracts, etc.) and are also known to interact with microbiota. Polyphenols’ biological activity is not limited to their antioxidant capacity: Indeed, they exert other effects, which may be beneficial in supporting sports practice. For example, pomegranate polyphenols are renowned for their anti-inflammatory and anti-infective activity [138].

Once taken orally, polyphenols have been shown to interact with microbiota at different levels [139]. For example, flavonoids can affect and reshape the composition of intestinal flora exerting prebiotic and bactericidal effects and—although the evidence is not conclusive—their systemic anti-inflammatory effects could be at least in part associated with the modulation of microbiota [140,141]. The ‘prebiotic-like’ effects of polyphenols have been observed using both in vitro studies with human gut microbiota, and in vivo in preclinical and clinical trials where supplemental polyphenols and polyphenols-rich foods were shown to modulate gut microbiota, stimulating the growth of beneficial bacteria such as *Lactobacilli* and *Bifidobacteria* [139]. Other beneficial species whose growth is favored by polyphenols include *Akkermansia*, *Faecalibacterium prausnitzii*, and *Roseburia* spp. [142,143]. In consideration of the above-mentioned positive interactions between polyphenols and gut microbiota, Marchesi et al. [121] introduced the concept of the “three P’s” for gut health: The three P’s stand for probiotics, prebiotics, and polyphenols. Hence polyphenols were promoted at the same biological level as prebiotics.

Unfortunately, many natural polyphenols, such as condensed or hydrolysable tannins and glycosylated polyphenols derivatives (bound with sugars such as glucose, galactose, rhamnose, ribulose, arabinopyranose, and arabinofuranose) are characterized by low gut absorption in humans. The reduced oral bioavailability represents a severe limit to the potential beneficial effects of these compounds [144]. Interestingly, these polyphenols, which usually remain inactive in the diet, are biotransformed to active compounds after removal of the sugar moiety by gut microbiota [2]. These metabolites may retain parental compounds’ antioxidant and pleiotropic activity, whilst also exhibiting increased gut absorption and better bioavailability. Hence, flavonoids, through the biotransformation operated by microbiota, can more easily reach the bloodstream and exert their biologically-relevant effects at the systemic level [145].

With regard to these effects, the traditional point of view of the health benefits attributed to the polyphenol fraction of foods and herbal supplements should be redirected towards their absorbable gut microbiota metabolites.

On the whole, it can be concluded that the reciprocally positive interactions between microbiota and polyphenols might converge toward the promotion of beneficial conditions supporting athletes in sport practice.

### 5.2. Probiotics

Probiotics are live non-pathogenic microorganisms, which, when administered in adequate amounts, confer microbial balance, particularly in the GI tract [146]. They have received a renewed interest in the past years in athletes, since they can contribute to general health and, indirectly, sustain and/or improve performance. Probiotics have been demonstrated to lower the frequency and duration of diarrhea and to stimulate humoral and cellular immunity [147]; furthermore, they show significant antioxidant activity both in vivo and in vitro [148,149]. As previously discussed, immune perturbations caused by heavy training and competition, increase susceptibility to URTI and impact the GI, and hence greatly affect performance [150]. Probiotics are effective in decreasing susceptibility to URTI and in reducing their incidence and/or their duration or severity [115,116,151] depending on factors such as the type of sport, athletes’ training status, and kind and duration of supplementation [152]. These effects can be explained by modulation of serum cytokines and secretory immunoglobulin A (SIgA), and by changes in number and activity of innate and adaptive immune cells [116,153]. Regular consumption of probiotics can modify intestinal epithelial cell proliferation and reduce permeability, increasing the expression of mucin genes, and mucin and antimicrobial peptide secretion, enhancing the barrier function of the mucosa [154]. Lamprecht et al. [155] analyzed the effects of a multi-species probiotic supplementation on markers of the intestinal barrier, oxidation and inflammation, at rest and after intense exercise in a randomized, double-blinded, placebo-controlled trial. They found that zonulin, a marker of enhanced gut permeability, decreased to normal range after supplementation for 14 weeks, and TNF-α and exercise-induced protein oxidation were also positively affected. Thus, probiotic supplementation can modulate intestinal barrier function, redox homeostasis, and low-grade inflammation after sustained exercise.

Roberts et al. [156] examined the effect of a multistrain probiotic/prebiotic/antioxidant intervention for 12 weeks preceding a long-distance triathlon on endotoxin unit levels and GI permeability in recreational athletes. They divided subjects into three groups: One group received a probiotic/prebiotic/antioxidant supplement (LAB^4^ANTI), one group received a pre/probiotic supplement only (LAB^4^), and the third group was a placebo; values were assessed at baseline, pre-race and six days post-race. The pre/probiotic supplementation (LAB^4^ and LAB^4^ANTI) significantly reduced endotoxin levels six days after the race, but only LAB^4^ANTI was effective pre-race. Even if faster times were registered in both supplemented groups, there was no statistically significant difference in mean race times. The authors concluded that “combined multistrain pro/prebiotic use may reduce endotoxin unit levels, with LAB⁴ANTI potentially conferring an additive effect via combined GI modulation and antioxidant protection”.

Investigations have revealed that *Lactobacillus* spp. has various biological effects, for instance, *Lactobacillus rhamnosus CNCMI–4317* was able to regulate cellular function and maintenance, lymphoid tissue structure and development, and immune system response [157]; furthermore, *Lactobacillus* spp., producing lactic acid, lead lactate-utilizing bacteria to produce butyrate [158].

Recently, the possible ergogenic and anti-fatigue effects of probiotics have been investigated. *L. plantarum* TWK10 has been shown to promote an increase in muscle mass, performance, and resistance to fatigue in supplemented mice [159]. In a human study, Huang et al. [160] analyzed the effect of six weeks TWK10 supplementation during exhaustive exercise. The probiotics significantly improved endurance performance and glucose content, suggesting that TWK10 can be beneficial to energy harvest. Thus, in addition to the well-documented antioxidant action, supplemental TWK10 might have an ergogenic anti-fatigue function [161,162]. However, few studies have focused on *lactobacilli* (probiotic) and their interaction with gut microbiota, and the possible ergogenic anti-fatigue function. The previously described results of Scheiman et al. [60] on *Veillonella* effects on athletic results can suggest a possible use of these strains as a supplement to improve performance. In a similar direction, Soares et al. [163] recently highlighted the benefits to the host metabolism resulting from *Saccaharomyces boulardii* (Sb) supplementation. In this study, the authors evaluated the effect of Sb supplementation on the rate of oxygen consumption (VO_2_), mechanical efficiency, and aerobic performance of Wistar rats subjected to fatiguing, incremental-speed exercise, and found that supplemental Sb does not affect resting aerobic metabolism, but significantly increases VO_2_ max and aerobic performance.

Among the most popular probiotics, kefir (KF), a drink rich in lactic ferments obtained from the fermentation of milk, has been proposed against hypertension, GI diseases, allergies, ischemic heart disease [164,165], fatigue and fatigue-related metabolites [166,167], and exercise-induced immune suppression [168]. KF is able to lower plasma lactate, ammonia, and creatine kinase (CK) levels, increasing the exercise performance and counteracting physical fatigue in mice [169], altering gut microbiota composition.

Interestingly probiotics are known to promote an improvement of mood state and disorders [170] and, based on literature [171], it is conceivable that the probiotic-mediated improvement of mood status may indirectly and positively impact on athlete’s conditions. The mechanisms whereby microbiota can influence mood, and more generally, CNS, have been comprehensively reviewed by Bravo et al. [172]. Although there are very few studies specifically dealing with this issue, based on the prevalence of animal studies, some authors reported beneficial effects of gut microbiota health on mood disorders which impact on performance, such as anxiety and depression [173,174,175,176]. The effects of a six-month administration of a probiotic multivitamin preparation in adults suffering from stress or exhaustion were studied by Gruenwald et al. [174]. The results showed a significant improvement in the general conditions of those who participated in the study, including a 41% improvement in perceived stress. Messaoudi et al. [175] investigated the similar-anxiolytic effects of *Lactobacillus helveticus* and *Bifidobacterium longum* supplementation on healthy human volunteers. The authors reported that a 30-day supplementation regimen promoted beneficial psychological responses. Rao et al. [176] investigated the effects of probiotic intake on anxiety and depression in chronic fatigue syndrome patients: Eight-week consumption of *Lactobacillus casei* resulted in a significant reduction in anxiety scores as compared to a placebo group. Finally, a multispecies probiotic supplementation was reported to positively affect the cognitive reactivity to a sad mood [174]. Although the participants in this study had no diagnosis of mood disorders, those who consumed the probiotic over four weeks significantly reduced their cognitive reactivity to sad mood, i.e., a condition that may favour progression into clinical depression. As to animal studies, although more research work is still needed, similarly to humans, there is some evidence indicating that gut microbiota health and composition affect animal depressive symptoms and behavior [177,178,179]. On the whole, the results of the relatively few animal and human studies suggest that the use of probiotics might be beneficial to mood, particularly in individuals with a history of mood disorders. In a different direction, there are also studies showing a correlation between physical activity and stress levels, with physical activity generally promoting beneficial effects on mood and affective state [180,181]. From a biochemical perspective, a positive correlation between physical exercise and the levels of tryptophan was described by Gostner et al. [182]: The authors suggested that physical exercise allows for increased levels of tryptophan which—once taken-up by the brain—may boost the synthesis of serotonin, subsequently leading to an increase in mood state. On the whole, it is conceivable that combining probiotic supplementation to maintain good microbiota conditions with an appropriate level of physical exercise may have additive—if not synergistic—beneficial effects on mood.

### 5.3. Proteins

Athletes are subjected to multi-stress conditions of regular, intensive, and/or prolonged exercise routines; to promote protein synthesis and the consequent adaptations to training, they need an adequate protein intake. Proteins should be consumed before and after the performance and regularly during the day to guarantee an efficient supply of essential amino acids [183], especially when increasing intensity and duration of the training session and competitions.

To clarify the impact of proteins on gut microbiota composition and functionality, protein quantity, quality, processing history (which impacts on protein digestion, presentation, and overall function), and source must be taken into consideration [184]. Protein sources with a different amino acid composition can impact differently on gut microbiota. Some studies suggest that plant-derived proteins are more useful than animal-derived proteins for microbiota, and consequently, for host metabolism. A high animal protein consumption has been linked to the *Bacteroides* enterotype [7].

Beaumont et al. [185] studied the effect of a three-weeks isocaloric supplementation with casein, soy-protein, or a maltodextrin compound in overweight humans. They reported that the high-protein diets had no effect on the bacterial composition and diversity of fecal microbiota. However, interestingly, both high-protein diets caused a shift of fecal and urinary metabolite (amino acid-derived isobutyrate, microbiota-host cometabolites indoxyl sulphate, and phenylacetylglutamine) concentration produced by gut microbiota. A decrease in fecal butyrate in both high-protein groups was found, suggesting a possible negative effect of a high-protein intake on colonic health. The two high-protein diets also led to an increase in the concentration of amino acid-derived bacterial metabolites, due to higher protein degradation by the gut microbiota.

Moreno-Perez et al. [186] evaluated the effect of 10-week protein supplementation (10 g of whey isolate plus 10 g of beef hydrolysate protein per day) on gut microbiota in recreational endurance athletes. Although there was no effect of SCFAs, ammonia, or faecal pH, they reported a decrease in health-promoting bacteria (*Roseburia*, *Blautia*, and *Bifidobacterium longum*); based on these data, the authors concluded that long-term protein supplementation might harm the gut microbiota of athletes. The effects of long-term high-protein supplement consumption on intestinal microbiota and amino acid fermentation have not been further investigated in recreational sports people and lifestyle users of protein supplements.

Microbial diversity has been found to positively correlate with protein intake and exercise in elite athletes [63], and thus, exercise training is suggested to be essential for healthy and balanced gut microbiota, and muscle mass and function. According to these observations, well-balanced protein intake should support not only athletic performance but also the well-being of the gut [187]. Although the physiological dietary protein requirements are elevated for athletes, high-protein diets may affect the gut microbiota and protein fermentation levels [94], with consequences for health [187]. Effects of protein supplementation for microbiota in athletes should be taken into consideration since nutritional recommendations regarding protein consumption in this population are higher than for the general population. The long-term consequences of the decrease in these bacterial taxa (*Blautia*, *Roseburia*, and *Bifidobacterium longum*) for gut health are unknown [186].

## 6. Sports Supplements with Moderate/Emerging Evidence of Interactions with Microbiota

### 6.1. Branched Chain Amino Acids (BCAAs)

Studies regarding the influence of BCAA supplementation on gut microbiota are still limited to animal studies, where several beneficial effects have been documented in pigs and mice [188,189,190,191,192]. Recently, it has been reported that dietary supplementation with BCAAs promotes intestinal development, enhances enterocyte proliferation, increases intestinal absorption of amino acids (AA) and glucose, and improves the immune defenses of piglets [193]. Yang et al. [194] reported an increased abundance of *Akkermansia* and *Bifidobacterium* and a decreased level of *Enterobacteriaceae*, together with changes in lipid and sugar metabolism in BALB/C mice supplemented with a BCAA-enriched mixture (1.5 mg/g body weight per day, beginning at the 11th to the 15th month of age). The *Akkermansia* genus has been reported to reduce metabolic disorders and insulin resistance by reversing the effects of high fat diets, and by enhancing intestinal levels of endocannabinoids controlling inflammation, as well as enhancing the intestinal barrier and intestinal peptide secretion [195]. However, since the above data have been obtained in animals, specific studies are needed to understand the effects of BCAA supplementation on gut microbiota in humans.

### 6.2. Glutamine

Glutamine is the most abundant amino acid in human blood, skeletal muscle, and the free amino acid pool [196]. At the intestinal level, it promotes enterocyte proliferation, regulates tight junction proteins [197], improves intestinal barrier function and reduces intestinal inflammatory response [198]. Glutamine is widely used in sports nutrition; in particular, its supplementation seems to increase muscle glycogen synthesis, reduce ammonia accumulation induced by exercise, and attenuate markers of muscle damage [199]. Analyzing the intervention of oral intake of 30 g per day of L-glutamine for a short period (14 days) in overweight and obese adults, de Souza et al. [200] noticed some alteration in the composition of gut microbiota. First, the authors reported a reduced *Firmicutes:Bacteroidetes* ratio compared to the control group; moreover, the number of bacteria from the *Veillonella* genus decreased, whilst that of *Prevotella* genus increased. To this regard, it has been reported that an increased amount of *Veillonella* is associated with higher levels of gut inflammation and the development of colitis and Crohn’s disease [201,202]; on the contrary, an increased the abundance of the *Prevotella* genus has been described as a shield against inflammation and non-infectious diseases of the colon [132]. Furthermore, a recent study by Abboud et al. [203] showed that 14 days of 30 g of glutamine supplementation induced a significant diminution in the waist circumference and circulating LPS levels in overweight subjects, and reduced the insulin levels in obese subjects. These findings suggest that glutamine supplementation in humans may promote a healthier microbiota composition with anti-inflammatory implications; the latter probably due to the decrease of *Veillonella* and increase of *Prevotella* genera. However, although these beneficial effects are well-documented in studies carried out on animals [204], the literature in humans is still scarce, making it necessary to develop more studies.

### 6.3. Sodium Bicarbonate

Sodium bicarbonate is a cheap and widely available household product. The consumption of bicarbonate-rich mineral water is used as a sport supplement to improve performance [205]. This supplement, taken before training or competition, is reputed to enhance intracellular and/or extracellular buffering capacity by managing the progressive increase in intracellular acidosis during intense exercise [206]. Microbiome analysis demonstrated that the consumption (500 mL per day, for 7 days) of bicarbonate-rich mineral water (ca. 2.5 g/l of bicarbonate ions) might promote a reshape of microbiota, increasing lean-inducible bacteria (*Christensenellaecae* and *Dehalobacteriaceae*) and decreasing *Bifidobacteriaceae* [207]. Interestingly, higher *Christensenellaceae* abundance has been previously reported in lean subjects (BMI < 25) as compared to obese ones (BMI > 30) [208].

### 6.4. Vitamin D

Vitamin D is known to significantly contribute to skeletal mineralization regulating calcium and phosphorus-homeostasis, and promoting endocrine effects on bone, intestine, parathyroid glands and kidney [209]. There is also growing evidence that Vitamin D regulates many other cell functions, and its potential impact on skeletal muscle mass and strength is receiving more considerable attention. The biological actions of vitamin D on muscle cell differentiation, metabolism, and function may be multiple, acting through direct and indirect, genomic and non-genomic pathways [210]. Furthermore, athletes may exhibit vitamin D deficiency, particularly during the winter in northern countries [211]. For these reasons, vitamin D supplementation is one of the most popular in sports nutrition. An additional and emerging rationale for its supplementation might be that vitamin D deficiency negatively affects the intestinal microbiome, hampering its capacity to produce B vitamins in the intestine, whose reduced availability may negatively impact the immune system [212].

An experimental study conducted on healthy volunteers [213] showed that high-dose (≥34,300 IU/week for eight weeks) intakes of oral vitamin D caused, in the upper gastrointestinal tract, a decline in *Proteobacteria* and an increase in *Bacteroidetes*, and in general, in bacterial richness. In parallel, a decrease of opportunistic pathogens like *Pseudomonas* and *Escherichia/Shigella* was observed. More recently, a comprehensive review [214] found evidence in the literature indicating that vitamin D influences the composition of the gastrointestinal microbiome, supporting that vitamin D supplementation could be useful in healthy subjects and hence, also in athletes. However, given the paucity and heterogeneity of the studies conducted so far, and the emerging importance in health and disease of the relationships between vitamin D and the microbiome, further and specifically designed studies and trials are warranted.

### 6.5. Omega-3 and Polyunsaturated Fatty Acids (PUFAs)

Polyunsaturated fatty acids (PUFAs) are known to maintain the fluidity of cell membranes and regulate cell signaling, gene expression, and cellular function, also serving as substrates for the synthesis of lipid mediators. The effects of PUFAs on gut microbiota are not well defined. However, experimental studies suggested that omega-3 PUFAs work with host immune cells to ensure intestinal wall integrity; furthermore, they can work with microbiota in regulating inflammation and the immune system [215,216]. Omega-3 PUFAs can exert anti-inflammatory effects post-exercise, interacting with prostaglandin synthesis [217], and can affect gut integrity by improving epithelial barrier function in animal models [218]. They have also been shown to decrease the *Firmicutes:Bacteroidetes* ratio and the levels of *Coprococcus* and *Faecalibacterium*; increasing the abundance of *Bifidobacterium*, *Lachnospira*, *Roseburia*, and *Lactobacillus* and other butyrate-producing bacterial genera [219,220,221]. Furthermore, omega-3 PUFA plasma levels correlate with the *Lachnospiraceae* family, a SCFA-producing bacteria [219]. Neuropsychiatric disorders and dysbiosis induced by social instability stress during adolescence were prevented by supplementation with omega-3 and the protective effect was long-term maintained through adulthood [222,223]. Since omega-3 PUFAs are known to modulate the gut microbiota composition, they have been recently proposed as prebiotics [224].

### 6.6. Carbohydrate-Electrolyte Sport Drinks

Carbohydrate sports drinks provide worthwhile benefits to endurance performance compared with noncaloric control beverages [225]. Carbohydrates in different formulations (liquid, gels, or solid) are frequently associated with electrolytes. Moreover, some authors reported that these supplements could increase gut discomfort depending on the intestinal absorption, formulation, and or/the co-presence of specific components [226]. However, there are no studies aimed at highlighting changes in the abundance of the different bacterial strains of intestinal flora compared to the different formulations of carbohydrate (CHO)-sport drinks, therefore specific studies are needed to clarify their interactions with microbiota.

### 6.7. L-Carnitine

L-carnitine-containing supplements are popular and widely used by athletes to increase physical performance [227]—due to its role in mitochondrial fatty-acid oxidation—and to facilitate recovery from exercise [228]. Carnitine may be absorbed both in the small and in the large intestine. The gut microbial population may influence the absorption rate of carnitine by the large intestine: Indeed, a high intestinal bacterial population can inhibit carnitine absorption. Carnitine plays a role in maintaining the ability of the colonic microbiota of fiber fermentation; furthermore, low dietary fiber intake can affect the metabolic carnitine pathways that have been linked to an increased risk of atherosclerosis and cardiovascular diseases [229]. Indeed, L-carnitine might be converted into atherosclerosis- and thrombosis-promoting metabolites via gut microbiota-dependent transformations [230,231,232]; the extent of this transformation is dependent on the diet habit, i.e., omnivorous > vegan/vegetarian diets. However, these results should be taken with caution due to the low sample size in these studies. On the other hand, carnitine may instead have a beneficial effect on metabolic health and cardiovascular function via stimulation of glucose oxidation, improved glucose tolerance, and insulin sensitivity [230].

### 6.8. Caffeine

Caffeine consumption is very popular among sports practitioners due to its supposed beneficial effects on performance. Indeed, caffeine, acting as a competitor for adenosine in CNS, is reputed to hamper the negative effects that adenosine causes on neurotransmission, excitation, and pain perception [233]. However, although caffeine supplementation is widely utilized by athletes and there is vast literature on its biological and pharmacological effects, its influence on gut microbiota is not well known. Caffeine can be taken in various and very normal ways as it is contained in coffee, multiple drinks, extracts, and foods. To date, there are few studies on the possible effects of caffeine intake on the composition of gut microbiota. For instance, Jaquet et al. [234] found that a daily dose of three cups of coffee (over three weeks in 16 healthy adults) promoted an increase in the number of the reportedly beneficial *Bifidobacteria* strains. However, as declared by the authors themselves, this study was affected by some limitations, such as the absence of control and placebo. More recently, Janssens et al. [235] found that Green Team (GT) supplementation (corresponding to a daily intake of 0.27/0.45 g caffeine) for 12 weeks did not have a significant effect on the composition of the gut microbiota; no significant differences in the fecal bacterial diversity and community structure were observed between treatment (green tea or placebo) and time (baseline and week 12) for the measured variables. Therefore, they concluded that catechin- and caffeine-rich GT supplementation has no long-term effects on the composition of the gut microbiota in healthy normal-weight and overweight subjects.

However, the main limitation of both studies, which gave apparently conflicting results, is that they investigated the effects of products (coffee or green tea dry extract) containing not only caffeine, but also a complex mixture of other bioactive compounds such as polyphenols. Hence, to date, although caffeine is a prominent ingredient in many popular sports supplements, it is not yet possible to establish if it may produce any significant effect on microbiota composition.

## 7. Conclusions

In summary, intensity, timing, and type of exercise can influence the composition of gut microbiota, as it also relates to gender, age, health, and training status of subjects. It has been demonstrated that physical activity performed at low levels, but continuously, can increase microbiota diversity, improving the metabolic profile and immunological responses of the subjects, while acute strenuous exercise may cause deleterious effects on the athletes’ microbiota and his general health.

**Figure 1:**
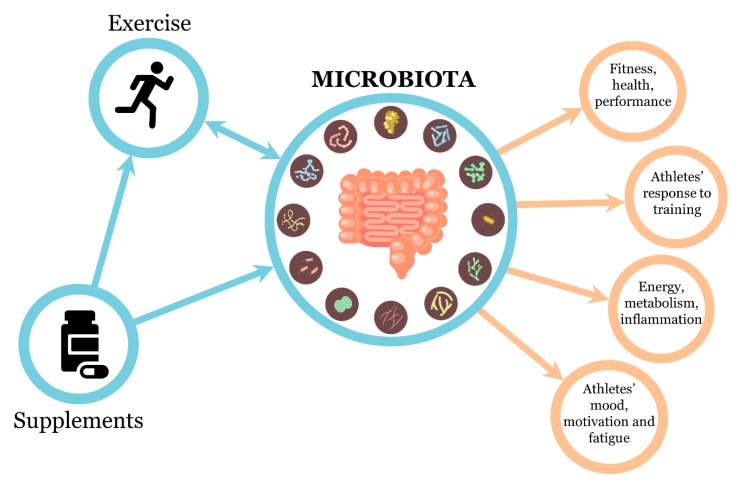
Beneficial effects of exercise, supplements, and microbiota interactions on athletes’ health and performance.

Gut microbiota composition can influence training adaptation and athletic performance: Indeed, it is involved in the metabolism and delivery of nutrients, hormones, and vitamins important to sustain exercise. Furthermore, specific gut bacterial species can be an aid for athletes by producing beneficial metabolites, such as the antioxidant bioactive molecules, SCFAs, which may help to improve metabolic, immune and barrier function, and anti-inflammatory and antioxidant systemic effects. On the whole, good gut microbiota conditions positively affect athletes’ health with beneficial consequences on their training adaptation and performances (Figure 1).

Some supplements intended for sport use contain probiotics, proteins, polyphenols, and other compounds which are known to interact with microbiota at multiple levels, for example in promoting the selection and growth of healthy specific microorganisms, such as *Bifidobacteria* and *Lactobacillus,* and at the same time, limiting the bacterial species that produce toxic metabolites.

To this end, the design of methods, including adequate and rational supplementation, capable of implementing the richness and metabolic capacity of the microbiota could be a useful strategy to improve athletes’ health, training response, adaptations, and ultimately, performance. Disappointingly, the interactions and interplay between exercise, gut microbiota, and sport nutritional supplements are still largely unknown, and should be a research focus in the near future.

## Figures and Tables

**Table 1:** Exercise and microbiota.

Reference	Models	Sample Size	Type of Exercise	Duration of the Intervention	Effects on Microbiota and Metabolic-Functional Responses
[75]	Wistar rats	14 (*n* = 7/group)	Voluntary wheel running vs. control	5 weeks	↑ Butyrate-producing bacteria; ↑ butyrate
[76]	Sprague-Dawley rats ABA group; control ABA group	40 (*n* = 10/group)	Voluntary wheel running. (i): rats submitted to the same food restriction schedule as ABA with no wheel access exercise, (iii) exercise group: rats feed ad libitum with free access to the activity wheel, and (iv) ad libitum group: rats feed ad libitum but without access to the activity wheel	6 days	↑ *Bacteroidetes*; ↓ *Firmicutes*; ↑ diversity; ↓ *Bacteroides*; ↑ *Lactobacillus*; ↑ organic acid lactate, butyrate
[63]	Humans	86 (*n* = 20–23/group)	Male rugby players vs. healthy male controls	1 sampling point	↑ Diversity in athletes vs controls; ↓ *Firmicutes,* ↓ *Ruminococcaceae,* ↓ *Succinivibrionaceae*, and ↓ *Succinivibrio groups*; ↑ *Akkermansiaceae* and ↑ *Akkermansia* in low BMI athletes and controls; ↑ protein intake; ↑ CPK in athletes
[12]	Wild-type mice	48 (*n* = 12/group)	Voluntary wheel running. (i) LF/Sed, (ii) LF/Ex, (iii) HF/Sed, and (iv) HF/Ex	12 weeks	↓ *Firmicutes:Bacteroidetes* ratio; ↑ *Bacteroidetes* phylum; ↓ *Firmicutes* in a manner that was proportional to the distance run in mice fed with HFD ↓ *Actinobacteria*; exercise prevented DIO
[11]	Wild-type mice, DIO mice	40 (*n* = 10/group)	Motorized wheel.	16 weeks	↓ *Bacteroidetes*; ↑ *Firmicutes*; ↑ Cognitive abilities despite HFD
[78]	Humans	1493	Voluntary exercise (i) never, (ii) rarely, (iii) occasionally, (iv) regularly, and (v) daily	1 sampling point	↑ *Faecalibacterium prausnitzii*; ↑ α-proteobacteria diversity
[79]	Obese (Zucker), hypertensive (SHR) and Wistar rats	9 (*n* = 3/group)	Forced treadmill running (i) obese fa/fa (homozygous); obese (Obese rats), (ii) hypertensive, and (iii) Wistar-Kyoto rats with high blood pressure	4 weeks	↑ *Firmicutes;* ↓ *Proteobacteria;* ↑ *Lactobacillus;* ↑ *Allobaculum* (Hypertensive rats); ↑ *Pseudomonas* and *Lactobacillus* (Obese rats); ↑ *Clostridiaceae* and *Bacteroidaceae* families and *Oscillospira and Ruminococcus* genera ↔ blood lactate accumulation
[77]	C57BL/6J mice	30 (*n* = 9–10/group)	Voluntary wheel running vs. forced treadmill running or control	6 weeks	↓ Bacterial richness; ↔ *Bacteroidetes,* ↔ *Firmicutes* in both groups. ↓ *Turicibacter* in Voluntary group
[80]	Wild-type mice	24 (*n* = 8/group)	HIIT	6 weeks	↓ *Firmicutes:Bacteroidetes* ratio
[81]	GF-, SPF-, BF- gnotobiotic mice	24 (*n* = 8/group)	Swimming	To-exhaustion test	↓ Glutathione, ↓ catalase, ↓ SCFAs, in GF and BF vs SPF
[82]	Mice	38 (*n* = 9–10/group)	Low-intensity treadmill running type 2 diabetic db/db vs db/+ (heterozygote); or control	6 weeks	↓ Enterobacteriaceae and ↑ *Bifidobacterium* spp *Bifidobacterium* spp. in exercised non-diabetic mice ↓ *Bacteroides/Prevotella* spp. and *Methanobrevibacter* spp; ↑ *Lactobacillus* spp. and *Clostridium leptum* in both trained and untrained groups
[83]	Ovariectomized rats	15 (*n* = 7–8/group)	Voluntary wheel running vs. control	11 weeks	↔ *Bacteroidetes*; ↔ *Firmicutes:Bacteroidetes* ratio; ↓ *Firmicutes* in low-capacity running rats; ↑ *Firmicutes* in high-capacity running rats
[57]	F344 rats	40 (*n* = 20/group)	Voluntary wheel running; juvenile vs. adults	6 weeks	↑ *Bacteroidetes;* ↓ *Firmicutes*; ↑ bacterial genera in juvenile rats vs adults
[48]	Wild-type, DIO mice	36 (*n* = 18/group)	Voluntary wheel running vs. control	12 weeks	↑ *Faecalibacterium prausnitzii* in trained mice; ↓ Cox-2 in DIO trained mice
[85]	Wild-type mice	14 (*n* = 4/5/group)	Voluntary wheel running vs. forced treadmill running or sedentary	6 weeks	↑ Diarrhoea; ↑ IL-6; ↑ IL-1β; IL-17 colon gene expression; ↑ mortality in treadmill group; alleviated colitis symptoms; ↓ inflammatory gene in wheel group
[68]	Humans	39 (*n* = 14-12-13/group)	Observing levels of cardiorespiratory fitness	1 sampling point	↑ Diversity; ↑ butyrate-producing taxa in subjects with higher fitness
[84]	Humans	86 (*n* = 40–46/group)	Professional rugby players vs. control	1 sampling point	↑ SCFAs: acetate, propionate and butyrate, ↑ muscle turnover (fitness) in athletes vs. control group
[59]	Humans, healthy women	40 (*n* = 19–21/group)	Active women performing World Health Organization-recommended low dose of exercise vs. sedentary	1 sampling point	↑ *Faecalibacterium prausnitzii*; ↑ *Akkermansia muciniphila*; Gut microbiota composition ∝ body fat/muscular mass
[86]	C57BL/6J mice	42 (*n* = 10-11-21/group)	Voluntary wheel running vs. forced treadmill running or control	8 weeks	↔ Abundance in gut microbes, ↑ *Rikenellaceae* and *Lachnospiraceae*; ↔ host inflammatory response in both trained and control
[87]	C57BL/6J mice	16 (*n* = 7–9/group)	Forced treadmill running vs. control	4 weeks	↑ *Butyricimonas* and *Akkermansia*; improved cardiac function
[88]	Humans, BCS	12	Observing levels of cardiorespiratory fitness, anxiety, fatigue	3 months	Gut microbiota composition ∝ changes in cardiorespiratory fitness level and anxiety in BCS
[61]	Humans	33	Cyclists who reported either 6–10, 11–15, 16–20, or 20+ hours of exercise per week	1 sampling point	↑ *Bacteroides;* ↑ *Prevotella*; ↑ *Eubacterium*; ↑ *Ruminococcus,* and ↑ *Akkermansia*; ↑ *Methanobrevibacter smithii* archaeon had upregulation of genes involved in the production of methane
[89]	Humans, T1D, and healthy controls	20 (*n* = 10/group)	Male T1D vs healthy male controls, observing levels of physical fitness, glycemic control	1 sampling point	*Faecalibacterium* sp., *Roseburia* sp. and *Bacteroides* sp. were typically the most abundant members of the community in both patients with T1D and controls. Gut microbiota comparable between T1D-subjects in good glycaemic control + high physical fitness vs healthy control
[93]	Humans, premenopausal women	71 (*n* = 23–24/group)	Observing levels of cardiorespiratory fitness	1 sampling point	↑ *Bacteroides*; ↓ *Eubacterium rectale-Clostridium coccoides.* Gut microbiota composition ∝ cardiorespiratory fitness level. The association between VO_2max_ and *EreC*, however, appears to be mediated by body fatness
[70]	Humans	20 (16 males and 4 females)	Amateur runners	2 sampling points (one week before and one week after the half-marathon race)	Running did not affect the α-diversity, ↑ Coriobacteriaceae and Succinivibrionaceae *Coprococcus*, *Actinobacillus*, and *Ruminococcus bicirculans;* ↓ *Ezakiella* and *Romboutsia*
[9]	Mice	29 (*n* = 9–10/group)	Voluntary wheel running vs. forced treadmill running or control	6 weeks	Voluntary wheel running vs Forced treadmill running altered many individual bacterial taxa. ↓ *Turicibacter* spp. in VWR group
[90]	Humans, Overweight or obese	90 (*n* = 30/group)	Overweight or obese adults randomized to exercise-only, exercise + whey protein, or whey protein only groups	8 weeks	No significant changes in microbial species composition; ↑ bacterial diversity in to exercise-only, exercise + whey protein. Modest alterations of microbial metabolic potential
[91]	Humans	37 (*n* = 20 males, *n* = 17 females)	Observing levels of cardiorespiratory fitness	1 sampling point	↑ *Firmicutes:Bacteroidetes* ∝ VO_2max_
[92]	Humans	17	Sedentary, overweight women cycling exercise (low–moderate intensity)	6 weeks	↑ *Akkermansia*; ↓ *Proteobacteria*; ↓ Fructose and amino acid metabolism-related genes
[60]	Humans	38 (*n* = 26–12/group)	Marathon athletes vs. control	2 sampling points (one week before and one week after the race)	↑ *Veillonella atypica* converts lactate to propionate; ↑ Methylmalonyl-CoA pathway is overexpressed
[60]	Male C57BL/5	64 (*n* = 32/group)	Treated with *V. atypica* or *L. bulgaricus*	3 days. 2 sampling points, (before exercise and after exercise).	*Veillonella* inoculation improved treadmill performance; performance is improved in mice administered propionate via intracolonic infusion

Abbreviations: ↑ significant increase; ↓ significant decrease; ↔ = unchanged; ∝ = correlates; ABA = activity based anorexia; BF = *Bacteroides fragilis*; BMI = body mass index; BCS = breast cancer survivors; CPK = creatine-phosphokinase; DIO = diet-induced obese; Ex = exercise; GF = germ free; HIIT = high-intensity interval training; HFD = high fat diet; HF/Ex = high fat exercise; HF/Sed = high fat sedentary; LF/Ex = low fat exercise; LF/Sed = low fat sedentary; ND = normal diet; PBS = phosphate buffer saline; SCFAs = short chain fatty acids; Sed = sedentary; SPF = specific pathogen-free; SHR = strain hypertensive rats; T1D = Type 1 diabetes; VO_2_max = maximum rate of oxygen consumption.

**Table 2:** List of the most useful sport supplements able to provide benefits in terms of health and performance according to Close et al. [135].

Strong Evidence	Moderate or Emerging Evidence	Lack of Evidence
Antioxidants (Polyphenols) Probiotics Proteins	BCAA ^1^ L-Glutamine Sodium-Bicarbonate Vitamin D Omega-3 PUFAs ^2^ CHO ^3^-Electrolytes Sport Drink L-Carnitine Caffeine	Creatine Taurine Beta-Alanine Beetroot Juice Collagen Glucosamine Vitamin C

^1^ BCAA: Branched chain amino acids; ^2^ PUFAs: Polyunsaturated fatty acids; ^3^ CHO: Carbohydrates. Supplements are grouped based on their interaction with the gut microbiota: Strong (green), moderate/emerging (yellow), and lack of evidence (orange).

**Table 3:** Supplements and microbiota.

Supplement Category	Supplement	Reference	Models	Sample Size	Administration (Dose/Day and Duration) of Supplementation	Effects on Microbiome Taxonomy
Antioxidant	Flavonols (Quercetin)	[141]	Rats	23 Wistar rats (*n* = 5–6/group)	30 mg/kg BW/day for 6 weeks	↓ *Firmicutes:Bacteroidetes ratio*; ↓ *Erysipelotrichaceae*, ↓ *Bacillus*, ↓ *Eubacterium cylindroides*
	Anthocyanidins	[142]	Mice	36 mice (*n* = 12/group)	Cranberry extract (200 mg/kg) for 8 weeks	↑ *Akkermansia*
	Pomegranate Extract (Ellagitannin)	[143]	Humans	20 healthy subjects	1000 mg for 4 weeks	↑ *Actinobacteria; ↓ Firmicutes*
Probiotics	LAB; LAB-ANTI	[156]	Humans	30 triathletes subject (*n* = 10/group)	Two capsules in the morning for 12-week pre-race period and the six-day post-race period	↑ *Bifidobacteria* ↓ *Firmicutes* ↓ *Bacteroides*
	*Lactobacillus rhamnosus* CNCMI-4317	[157]	Mice	18 mice (*n* = 5-6-7/group)	11 days	*↑ Lactobacilli*
	*lactobacillus plantarum* TWK10	[159]	Mice	24 mice (*n* = 8/group)	LP10-1X:1 capsule 2.05 × 108 CFU/kg per day and LP10-5X: 1.03 × 109 CFU/kg for 6 weeks	*↑ Lactobacilli*
	*lactobacillus plantarum* TWK10	[160]	Humans	16 male adults (*n* = 8/group)	1 capsule per day for 6 weeks	*↑ Lactobacilli*
	*Saccaharomyces boulardii*	[163]	Wistar Rats	26 males (11 weeks old) (*n* = 13/group)	108 CFU·kg^−1^·day^−1^ for 10 days	↑ *Saccharomyces* ↑ *Bacteroides* ↓ *Firmicutes* ↓ *Proteobacteria* ↓ *Tenericutes*
	Kefir	[169]	Mice	32 mice (*n* = 8/group)	1 -vehicle group 2 -KF-1X, (2.15 g/kg/day) 3 -KF-2X, (4.31 g/kg/day) 4 -KF-5X, (10.76 g/kg/day) for 28 days	KF-1X: ↑ *Ruminococcaceae* KF-2X: ↑ *Bacteroides* ↑ *Bacteroidia* ↓ *Firmicutes* ↓ *Clostridiales* ↓ *Clostridia* KF-5X: ↑ *Bacteroides* ↓ *Firmicutes* ↑ *Rikenellaceae*, ↑ *Bacteroidales*, ↑ *Bacteroidia* ↓ *Clostridia*
	*Probiotic formulation (PF): consisting of Lactobacillus helveticus R0052 + Bifidobacterium longum R0175*	[175]	Humans	55 subjects (*n* = 26–29/group)	1·5 g/d of PF, 1 time per day, for 30 days	↑ *Lactobacillus;* ↑ *Bifidobacteria*
	*Lactobacillus Casei*	[176]	Humans	39 Chronic Fatigue Syndrome patient (*n* = 16–19/group)	1 sachet, three time per day, 8 weeks	↑ *Lactobacillus;* ↑ *Bifidobacteria*
Protein	Isolated Soy Protein (SOY); Isolated Milk Protein (CAS); Control	[185]	Humans	38 overweight subjects (*n* = 13/group)	15% of participants’ habitual energy intake for 3 weeks	No effects on microbiota composition
	Whey Isolate + Beef Hydrolysate	[186]	Humans	24 endurance recreational athletes (*n* = 12/group)	10 g of whey + 10 g of beef protein for 10 weeks	↑ *Bacteroidetes* phylum; ↓ *Firmicutes* phylum; ↑ *Bacteroides* genus; ↓ *Citrobacter* genera; ↑ *Klebsiella* genera; ↓ *B. Longum*
BCAA	BCAA-enriched mixture	[194]	Mice	18 BALB/c male mice (*n* = 9/group)	1.5 mg/g BW for 4 months	↑ *Akkermansia*; ↑ *Bifidobacterium*; ↓ *Enterobacteriaceae*
Glutamine	L-Glutamine; L-Alanine	[200]	Humans	33 overweight/obese adults (*n* = 12–21/group)	30 g for 14 days	↓ *Firmicutes:Bacteroidetes* ratio; ↓ *Veillonella* genus; ↑ *Prevotella* genus
Sodium Bicarbonate	Bicarbonate-enriched water	[207]	Humans	19 healthy subjects	2.5 g/L in 500 mL for 7 days	↑ *Christensennellaceae* ↑ *Dehalobacteriaceae* ↓ *Bifidobacteriaceae*
Vitamin D	Vitamin D_3_	[213]	Humans	16 healthy subjects	980 IU/kg BW for 4 weeks, 490 IU/kg BW for 4 weeks	Upper GI tract: ↓ *Gammaproteobacteria* (↓ *Pseudomonas* spp.; ↓ *Escherichia/Shigella* spp.); ↑ Bacterial richness. No major changes in the terminal GI tract.
Omega-3	Mixed eicosapentaenoic acid/docosahexaenoic acid	[221]	Humans	22 healthy adults	4 g for 8 weeks	↑ *Bifidobacterium*, ↑ *Roseburia*, ↑ *Lactobacillus*
	Fish oil; Sunflower oil	[219]	Humans	132 infants (*n* = 60–72/group)	9 months	↑ *Bacteroidetes*
Caffeine	Instant coffee powder	[234]	Humans	16 healthy adults	3.4 g of coffee in 150–200 mL water, 3 cups/day for 3 weeks	↑ *Bifidobacterium* spp.
	Green tea extract	[235]	Humans	58 healthy adults (*n* = 28–30/group)	0.27–0.45 g/day of caffeine for 12 weeks	No effects on microbiota composition

Abbreviations: ↑ significant increase; ↓ significant decrease; BW = body weight; KF = kefir; BCAA = branched-chain amino acids; GI = gastrointestinal.
